# Enhancing pediatric attention-deficit hyperactivity disorder treatment: exploring the gut microbiota effects of French maritime pine bark extract and methylphenidate intervention

**DOI:** 10.3389/fnut.2024.1422253

**Published:** 2024-08-27

**Authors:** Anne-Sophie Weyns, Sarah Ahannach, Tim Van Rillaer, Tess De Bruyne, Sarah Lebeer, Nina Hermans

**Affiliations:** ^1^Natural Products and Food Research and Analysis – Pharmaceutical Technology (NatuRA-PT), Department of Pharmaceutical Sciences, University of Antwerp, Antwerp, Belgium; ^2^Laboratorium of Applied Microbiology and Biotechnology, Department of Bioscience Engineering, University of Antwerp, Antwerp, Belgium

**Keywords:** polyphenols, French maritime pine bark extract, attention-deficit hyperactivity disorder, methylphenidate, gut microbiome, prebiotics

## Abstract

**Introduction:**

The pathogenesis of Attention-Deficit Hyperactivity Disorder (ADHD) is thought to be multifactorial, with a potential role for the bidirectional communication between the gut microbiome and brain development and function. Since the “golden-standard” medication therapy with methylphenidate (MPH) is linked to multiple adverse effects, there is a need for alternative treatment options such as dietary polyphenols. These secondary plant metabolites exert antioxidant and anti-inflammatory effects, but much less is known about their impact on the gut microbiota. Since polyphenols are believed to modulate gut microbial composition, interventions might be advantageous in ADHD therapy. Therefore, intervention studies with polyphenols in ADHD therapy investigating the gut microbial composition are highly relevant.

**Methods:**

Besides the primary research questions addressed previously, this study explored a potential prebiotic effect of the polyphenol-rich French Maritime Pine Bark Extract (PBE) compared to MPH and a placebo in pediatric ADHD patients by studying their impact on the gut microbiota via amplicon sequencing of the full length 16S rRNA gene ribosomal subunit (V1-V9).

**Results:**

One interesting finding was the high relative abundance of *Bifidobacteria* among all patients in our study cohort. Moreover, our study has identified that treatment (placebo, MPH and PBE) explains 3.94% of the variation in distribution of microbial taxa (adjusted *p*-value of 0.011).

**Discussion:**

Our small sample size (placebo: *n* = 10; PBE: *n* = 13 and MPH: *n* = 14) did not allow to observe clear prebiotic effects in the patients treated with PBE. Notwithstanding this limitation, subtle changes were noticeable and some limited compositional changes could be observed.

**Clinical Trial Registration:**

doi: 10.1186/S13063-017-1879-6

## Graphical abstract

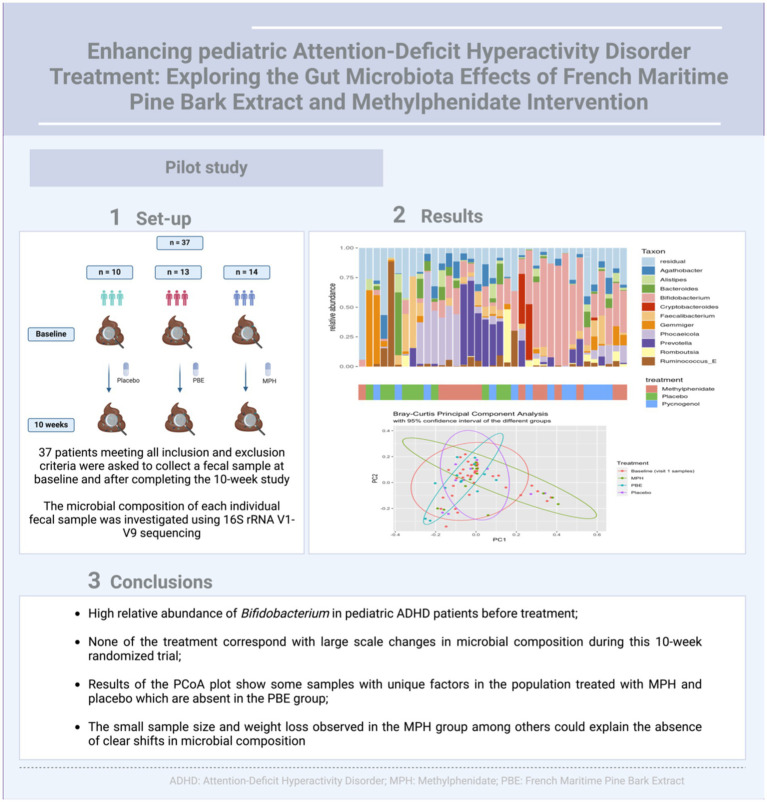

## 1 Introduction

Attention-Deficit Hyperactivity Disorder (ADHD) is the most prevalent neurodevelopmental disorder affecting around 8–12% children worldwide and often persists into adolescence and adulthood (1, 2). Although multiple parameters such as structural and functional central nervous system (CNS) abnormalities may be involved in ADHD pathophysiology, there is emerging evidence for immune and oxidant-antioxidant imbalances (3–6). In addition, it has been postulated that the ‘gut-brain’ axis is impaired in ADHD. This has been hypothesized based on the increasing number of some observational studies (n (studies): ≤ 9) (7) showing a link between intestinal function, gut microbiome and the CNS, suggesting that dysbiosis in the gut could be involved in the pathophysiology of ADHD (8). Unfortunately, this impairment of the gut-brain axis has not yet been well substantiated, with only limited intervention studies associating gut microbiome modulation with clinical benefits in this patient group (9, 10). Treatment with methylphenidate (MPH) is currently the first method of choice in ADHD therapy. However, it causes side effects such as loss of appetite and sleep problems and has a significant personal, social and financial burden for patients, while evidence on long-term efficacy is still lacking (11–14). Currently, little is known about the impact of psychostimulants such as MPH on the gut microbial composition (15). Given the new insights in the pathogenesis of ADHD, targeting the associated immune and oxidant-antioxidant imbalances or gut microbiome, could provide novel therapeutic treatment options.

French Maritime Pine Bark Extract (PBE; Pycnogenol®, Horphag Research), is a patented polyphenol-rich extract from the French maritime pine (*Pinus pinaster*) and is standardized to contain 70 ± 5% procyanidins (16, 17). Supplementation has been linked to various health benefits such as antioxidant activity and anti-inflammatory properties as suggested by both *in vitro* and *in vivo* research work (18–20). Some recent studies have also investigated PBE in the context of pediatric ADHD. A small randomized trial by Trebaticka et al. (21) already suggested a therapeutic benefit of PBE in ADHD and Weyns et al. (22) recently confirmed these findings. Moreover, present data suggest that dietary polyphenols could possibly act on the gut microbiota as prebiotics by specific stimulation of beneficial microbial species (e.g., enhancing the growth of bacterial families such as *Bifidobacteriaceae* and *Lactobacillaceae*) and decreasing the population of more harmful taxa (e.g., *Escherichia coli*, *Clostridium perfringens* and *Helicobacter pylori*) (23–25). According to the most recent consensus statement of the International Scientific Association for Probiotics and Prebiotics (ISAPP), a prebiotic is considered to be ‘a substrate that is selectively utilized by host microorganisms conferring a health benefit’ (26). For instance, after an intervention study in 22 healthy humans, Tzounis et al. reported increased abundance levels of *Lactobacillus* spp. and *Bifidobacterium* spp. after consumption of cocoa flavanols thereby indicating the potential prebiotic effects associated with flavanol-rich (flavonoids, class of polyphenols) foods (27). Another clinical study in 30 healthy male and female volunteers showed that consumption of blueberry products (especially rich in anthocyanins, an important subclass of flavonoids (28)) led to significantly increased abundances of *Lactobacillus* spp. and *Bifidobacterium* spp., which have been associated to multiple health benefits such as inhibition of gut pathogens, synthesis of vitamins and enhancing the immune system (29, 30). Also, consumption of red wine polyphenols significantly increased the number of *Bifidobacterium* spp. in the gut, while the quantity of *Lactobacillus* spp. was unaltered (31). Furthermore, Bacteroidetes and Firmicutes are the main short-chain fatty acid (SCFA)-producing taxa in the human gut and dysbiosis of these phyla may affect norepinephrine and dopamine biosynthesis, involved in ADHD pathophysiology, by alterations in SCFA levels (32). Generally, the ratio of Firmicutes to Bacteroidetes (F/B-ratio) has been suggested as an important index for health status, and alterations in this ratio have been linked to pathological conditions such as obesity (increased F/B-ratio) and inflammatory bowel diseases (lower F/B-ratio) (33–36). According to Wang et al. a trend toward a slightly higher F/B-ratio was observed in children with ADHD as compared to healthy controls (8). A diet rich in polyphenols can regulate the F/B-ratio (37). For example, earlier research by Yuan et al. demonstrated that a diet intervened with tea polyphenols rich in catechins resulted in an increase in the number of Firmicutes and a decrease in the number of Bacteroides (higher F/B ratio) (38). However, to the best of our knowledge, a potential prebiotic function of PBE and/or effect on F/B ratio has not yet been explored.

In this study, we aimed to investigate the impact of PBE and MPH on the gut microbial composition of pediatric ADHD patients in a 10-week intervention. We particularly aimed to examine whether PBE exerts a possible prebiotic effect on gut microbiota and whether MPH impacts the microbial composition of the gut. By elucidating the effects of PBE and MPH on gut microbiota, we intent to offer novel insights into ADHD treatment.

## 2 Materials and methods

### 2.1 Study background

The detailed study protocol was previously published in Verlaet et al. (NCT02700685 and EudraCT 2016–000215-32) (39). Pediatric ADHD patients (aged 6–12 years, 89% Caucasian), both diagnosed *de novo* and formerly treated, were included between September 2017 and November 2020 and randomized to one of the three treatment groups (placebo, PBE, MPH) (Supplementary file S1). Treatments included 1 or 2 oral capsules at breakfast with MPH (Medikinet® Retard, Medice GmbH, MPH modified release: 20 or 30 mg/day if < or ≥ 30 kg, resp.), PBE (Pycnogenol®, Horphag: 20 or 40 mg/day if < or ≥ 30 kg, resp.) or placebo (only microcrystalline cellulose and magnesium stearate). Fecal samples were collected from a participant subgroup (*n* = 37) at the start and at the end of the 10-week trial. Based on power calculations, 144 participants were to be included in the trial, of which a subgroup (*n* = 20 in each treatment group) were asked to collect a fecal sample at baseline and after 10 weeks. Nevertheless, due to the expiry of study capsules combined with poor inclusion during the covid-19 pandemic, the trial was ended with 88 participants of which 37 donated a fecal sample. Although the subgroups for the microbiome analyses were slightly smaller, they proportionally remained the same (37/88 instead of 60/144). The fecal donors all met the inclusion and exclusion criteria as mentioned in the study protocol (Table 1) with no extra specific requirements imposed prior to fecal donation (39). A validated Food Frequency Questionnaire (FFQ) consisting of 50 questions on different food groups was adopted to assess participants’ global dietary habits throughout the study (40).

**Table 1:** Inclusion and exclusion criteria as published in the trial’s protocol (18).

**Inclusion criteria**	**Exclusion criteria**
Age 6–12 years (both inclusive)	Diagnosis of autism spectrum disorder
ADHD diagnosis	IQ < 70, conduct disorder (CD), tics, dyskinesia, personal or family history of psychotic disorder, bipolar illness, depression, or suicide attempt
Responsible caregiver to provide information about the patient’s functional status	Chronic medical disorder or acute inflammatory disease; glaucoma, heart disease, high blood pressure, or peripheral vascular disease
Patient and responsible caregiver have a sufficient level of knowledge of Dutch	Use of MAO inhibitor 14 days before inclusion; use of clonidine, guanethidine, seizure medicine, antidepressants, blood thinners, blood pressure medication, or diet medication 3 months before inclusion
Written informed consent by the patient’s legally accepted representative	Use of nutritional supplements or any medication for longer than 1 week during 3 months before inclusion

ADHD, attention-deficit hyperactivity disorder; MAO, monoamine oxidase.

### 2.2 Sample collection

A subgroup of 37 patients (placebo: *n* = 10, PBE: *n* = 13 and MPH: *n* = 14) were asked to collect a fecal sample at the start and after completing the 10-week study using a Protocult collection container (Ability Building Center, Rochester, United States) and temporarily store them at −20°C until pick-up. The samples were kept at −80°C at the lab of Natural Products and Food Research Analysis-Pharmaceutical Technology (NatuRA-PT) until further analysis. All samples were pseudonymized according to General Data Protection Regulation (GDPR) regulations and stored in the University of Antwerp Biobank.

### 2.3 Microbial DNA isolation

Microbial DNA was extracted from all fecal samples (*n* = 74) using the FastDNA™ SPIN Kit (MP Biomedicals, Irvine, CA) according to manufacture instructions. The concentration of DNA isolates was quantified by a Qubit 2.0 Fluorometer with the dsDNA HS Assay kit (ThermoScientific, United States). DNA concentration in the samples varied from 10 to 450 ng/μL.

### 2.4 16S rRNA gene amplification, Pacbio library preparation and sequencing

Sequencing and library preparation was performed as described by the manufacturer (PacBio, USA) in the SMRTbell-prep-kit 3.0. In brief, the bacterial 16S rDNA specific primers 27F (5′-AGRGTTYGATYMTGGCTCAG-3′) and 1492R (5′-RGYTACCTTGTTACGACTT-3′) were used to amplify the bacterial full-length 16S rRNA gene (41). The KAPA HiFi Hot Start DNA Polymerase (KAPA Biosystems, United Kingdom) was used to perform 20 of the following cycles of PCR amplification: denaturation at 95°C for 30 s, annealing at 57°C for 30 s and extension at 72°C for 60 s. Amplified DNA from the fecal samples was then pooled in equimolar concentration. Barcodes were added during a second round of amplification with PacBio Barcoded Universal primers so that the amplicons could be multiplexed on seven SMRT cells. Sequencing was carried out on a PacBio Sequel machine with 2.1 chemistry. The raw PacBio sequencing data were translated into circular consensus sequence reads (CCS) using the SMRTLink v 10.2 with default parameters.

### 2.5 Data import and data filtering

Sequence reads were processed using the ampliseq pipeline (42) from nf-core (43) using the Nextflow computational workflow manager (44). The nfcore/ampliseq is a bioinformatics analysis pipeline used for 16S rRNA gene sequencing data. Processed data was loaded from the tidyamplicons folder, a package for R, developed in-house to handle amplicon data in a tidy manner (github.com/Swittouck/tidyamplicons). Data filtering was used to remove low quality or uninformative features in order to improve downstream statistical analyses. A first quality check was performed by comparing the output profiles of the positive controls (Mock communities, HM-783D, Bei Resources) to its datasheet to analyze the quality of the workflow. Next, non-bacterial amplicon sequence variants (ASVs) were removed from the dataset along with an ASV longer than the expected 1,500 basepairs.

### 2.6 Metadata import

During the clinical trial, several questionnaires (e.g., FFQ) were filled out by the participants (Supplementary file S2). Specific ADHD behavior such as inattention or hyperactivity was also assessed using an ADHD-Rating Scale (ADHD-RS) filled out by teachers. In addition, significant biomarkers including catalase (CAT), neuropeptide Y (NPY), immunoglobuline A (IgA) and G_2_ (IgG_2_) as outlined in Weyns et al. (45) were taken into account upon further statistical analyses.

### 2.7 Statistical analyses

The relative abundance level of taxa was analyzed for family and genus levels and the F/B-ratio was calculated. The α-diversity which outlines the microbial community in individual samples was calculated with the inverse Simpson index, taking into account the richness (number of taxonomic groups) and evenness (distribution of abundances of the groups) (46) (results of other α-diversity indices are denoted in Supplementary file S3). Assessment of β-diversity was performed by a dissimilarity matrix (Bray–Curtis dissimilarity) and results of the β-diversity estimates were visualized using principal coordinate analysis (PCoA). To analyze the association of several factors including behavioral scores, biomarkers, weight, sex and food intake on abundance levels of taxa, differential abundance analyses were also performed using the multidiffabundance R package which combines different differential abundance workflows (limma, lmclr and maaslin2) (47). This was done because not a single of the methods is flawless for the analysis of mixed samples such as the relative abundances retrieved in amplicon sequencing. Hence, only results with significance in two out of three methods were reported. The variation between samples in Bray–Curtis distance can be explained using 54 covariates with the Adonis function from the R package vegan. The samples were stratified by subject ID to account for repeated measures and 999 permutations were used to calculate the *p*-value. Afterwards, these *p*-values were adjusted for multiple testing using the Benjamini and Hochberg method. Taken together, these 54 covariates explained 75% of the variation in microbial composition of the participants. Numbers in the differential abundance analyses indicate the number of methods for which a significant (in this case adjusted *p*-value <0.01) was retrieved. In the PBE group, effect sizes smaller than 2 were left out to manage interpretability, however, in the MPH group all effect sizes were kept in the analysis since they were rather small. For other analyses a p-value <0.05 was considered to be statistically significant. Power calculations of the statistical test used were not carried out since these require simulations of the data, for which we would need to estimate the effect size and the variability of the data.

### 2.8 Data availability statement

Sequencing data generated in this study are available at the European Nucleotide Archive (ENA) at EMBL-EBI under accession number PRJEB65903.

## 3 Results

### 3.1 Gut microbiome of pediatric ADHD children is dominated by Bifidobacterium and Phocaeicola taxa

**Figure 1:**
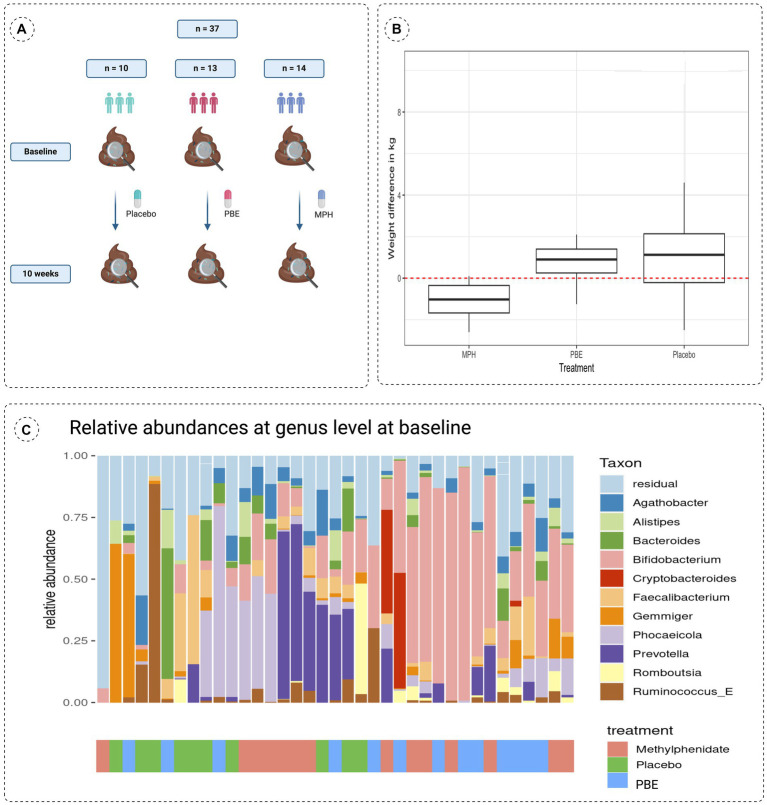
**(A)** Schematic overview of the study set-up showing a simplified timeline with collection times of fecal samples and the number of participants per treatment group. **(B)** Differences in weight observed after the 10-week study are compared to baseline (start of the study; red dotted line at zero) for each treatment group. **(C)** Stacked bar chart describing the baseline microbiome composition of all 37 participants of the 10 most abundant taxa at genus taxonomical level with their respective treatment groups indicated on the x-axis. MPH: Methylphenidate; PBE: French Maritime Pine Bark Extract.

Fecal samples of 37 ADHD patients (mean age: 10.3 years, 60% male) were collected at the start and after 10 weeks intervention with either PBE, MPH or placebo (Figure 1A). These treatment groups did not differ in demographic variables (e.g., age, height and weight) nor in dietary habits at the start of the study (data not shown). However, in Figure 1B a striking difference in weight (*p* = 0.0021) was observed in between the three groups after 10 weeks of treatment, namely a significant reduction of weight is noticeable in the group of participants treated with MPH. Looking at the microbial composition, Agathobacter (Firmicutes), Alistipes (Bacteroides), Bacteroides (Bacteroides), Bifidobacterium (Actinobacteria), Cryptobacteroides (Bacteroides), Faecalibacterium (Firmicutes), Gemmiger (Proteobacteria), Phocaelcola (Bacteroides), Prevotella (Bacteroides), Romboutsia (Firmicutes) and Ruminococcus_E (Firmicutes) showed to be the most dominant genera in our overall population (Figure 1C). Of note, Bifidobacterium and Phocaeicola spp. show a high relative abundance and prevalence in all baseline samples (>30% relative abundance in approximately 35 and 16% of the samples, respectively). Additional information on the core microbiome of included participants can be found in Supplementary file S4.

### 3.2 Differences in gut bacterial communities between the treatment groups

**Figure 2:**
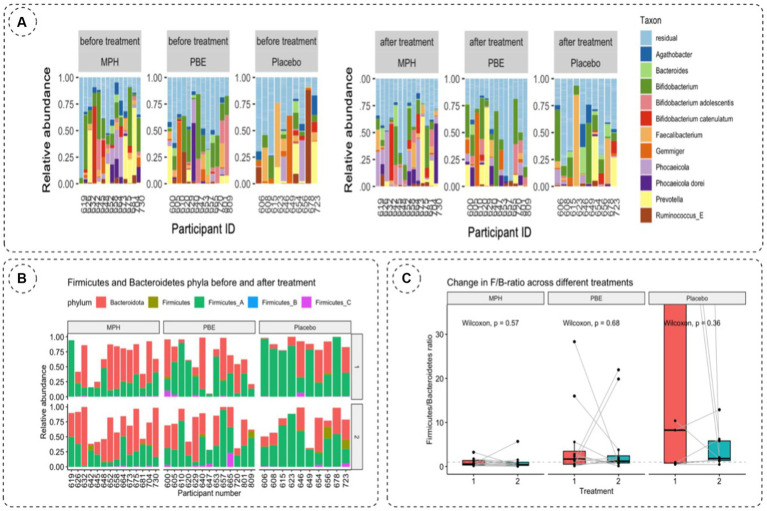
**(A)** Relative taxonomic abundances of 11 most abundant genera per participant based on 16S rRNA gene amplicon sequencing of fecal samples at baseline (visit 1) and after 10 weeks (visit 2) to evaluate the effect of treatment on the gut microbiome. **(B)** Relative abundances of Bacteroidetes and Firmicutes per participant and per study group on the first and second study visit. Bacteroidetes are depicted in red, Firmicutes are denoted ingreen, blue and pink stacked bars. **(C)** Change in F/B-ratio across the MPH, placebo and PBE group at baseline (vis1, red bar) and 10-weeks (vis2, bluebar). Statistical significance was tested using the paired Wilcoxon test. *p*-values are visualized on the figure. F/B-ratio: Firmicutes/Bacteroidetes ratio;MPH: methylphenidate; PBE: French Maritime Pine Bark Extract.

To investigate the impact of the three different treatments on the microbial composition of the gut between baseline and 10-week samples, the relative abundance of taxa at genus level was visualized per cohort (PBE, MPH and placebo; Figure 2A). Generally, no clear individual microbial shifts in relative abundance were observed after 10 weeks.

Since the literature often describes the balance between Firmicutes and Bacteroidetes and their impact on normal intestinal homeostasis (36) we also analyzed the relative abundances of Firmicutes and Bacteroidetes phyla and their ratios for each study cohort at baseline (visit 1) and after 10 weeks (visit 2) (Table 2). For the total Firmicutes relative abundance number, all Firmicutes phyla (Firmicutes_A, Firmicutes_B and Firmicutes_C) were summed. The F/B-ratio was then calculated for all samples containing Bacteroidetes (Figure 2B). A paired Wilcoxon test showed no significant trends in F/B ratio before and after treatment for the different treatment cohorts (Figure 2C).

**Table 2:** Overview of the relative abundances of Firmicutes and Bacteroidetes for each treatment group per study visit.

**Treatment**	**Visit**	**Mean F/B-ratio**	**Relative abundance Firmicutes**	**Relative abundance Bacteroidetes**
MPH	1	0.75	28.73%	38.22%
MPH	2	0.56	26.11%	46.48%
Placebo	1	2.68	65.29%	24.36%
Placebo	2	2.20	51.09%	23.26%
PBE	1	1.26	37.5%	29.72%
PBE	2	1.47	42.95%	29.12%

MPH, methylphenidate; PBE, French maritime pine bark extract.

**Figure 3:**
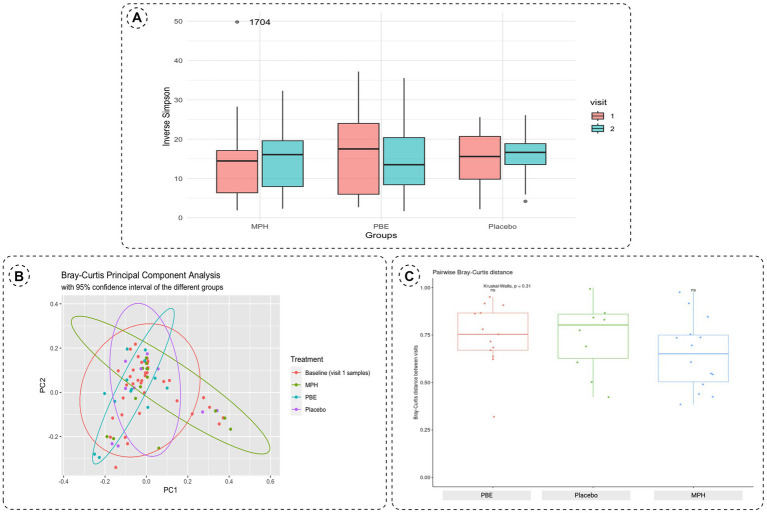
**(A)** Boxplots of the α-diversity between baseline (visit 1, red box) and 10-week samples (visit 2, blue box) for each study group using the inverse Simpson index. The outlier in the MPH group is represented by its corresponding participant ID. **(B)** Principal Component Analysis plot distributing the samples according to β-diversity measured with Bray-Curtis distances. Samples are colored by their respective group (orange: all baseline samples;green: 10-week samples MPH; blue: 10-week samples PBE and purple: 10-week samples placebo). **(C)** Boxplots of the pairwise Bray-Curtis distance of individuals at baseline and after 10-weeks per treatment group. Significance was tested using the Kurskal-Wallis test. Each boxplot represents the data range within the 1.5 interquartile range (IQR) with the median depicted as a horizontal line. Outliers are plotted as individual datapoints outside 1.5 times the IQR above the upper quartile and below the lower quartile. MPH, methylphenidate; PBE, French maritime pine bark extract.

To assess the within-samples diversity, the inverse Simpson index was calculated as depicted by Figure 3A. Though the α-diversity seems to decrease in the group treated with PBE, statistical testing with a Wilcoxon Ranked Sum test did not show a significant difference (*p*-value = 0.95) before and after PBE treatment (data not shown). Next, the Bray-Curtis distance matrix was calculated for the between-samples diversity and a PCoA was performed to assess the β-diversity (Figure 3B). Most samples from the different groups are scattered across the plot, but a cluster of 11 samples could be observed without any PBE treated participant’s samples. Nevertheless, the goodness of fit for the PCoA is only 8.8%, meaning that a representation of this large dataset in a limited 2D visualization is not optimal (results of the adonis2 function can be find under 3.3; line 260–265). Figure 3C illustrates the pairwise Bray-Curtis distance of individuals at baseline and after 10-weeks with their respective treatment. No significant difference between individuals from the active treatment groups as compared to placebo were observed, illustrated by the non-significant *p*-values.

**Figure 4:**
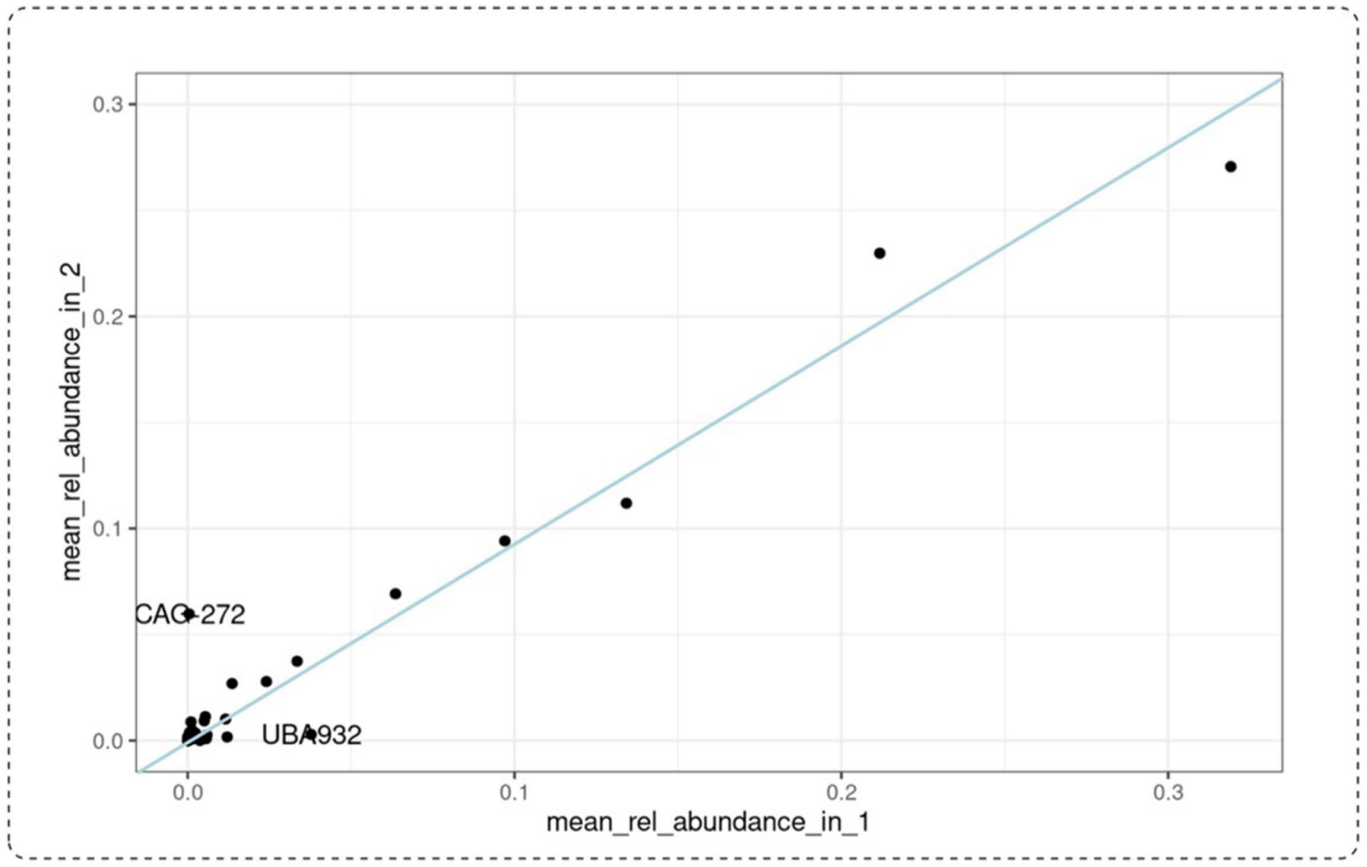
Mean relative abundance at taxa family level of patients receiving PBE for 10 weeks. Baseline visit is represented on the x-axis, 10-week visit on the y-axis. CAG-272: species belonging to Firmicutes. UBA-932: unculturable belonging to the Bacteroidales (Bacteroidetes). The blue line indicates samples with a taxonomic relative abundance after 10-week treatment similar to baseline samples. PBE, French maritime pine bark extract.

To investigate the potential prebiotic effect of PBE, we looked at the taxa family level of participants from the PBE cohort (Figure 4). One taxon was on average reduced in relative abundance after the treatment with PBE than after: *UBA932* (unculturable, Bacteroides). This unculturable bacterial strain was found in baseline and 10-weeks samples of patient 653 whereas for participant 720 it is found in relatively high abundance at the first study visit. However, since these changes in abundance occur only in two samples, this effect is most likely overrated. After treatment with PBE, there is another taxon that appears more abundant namely *CAG-272* (Firmicutes) in participants 629 and 657. Particularly in the latter the relative abundance after 10-weeks of PBE treatment is higher than compared to baseline. The rest of the datapoints follow an imaginary straight line depicted as a blue line in the graph (Figure 4) through the origin, thereby indicating that the relative abundance of taxa after 10-week treatment with PBE is similar to baseline. Due to the limited amount of datapoints, statistical testing would not indicate clear significant differences in taxa at family level for the PBE cohort.

### 3.3 Impact of host covariates on the gut microbiome

**Figure 5:**
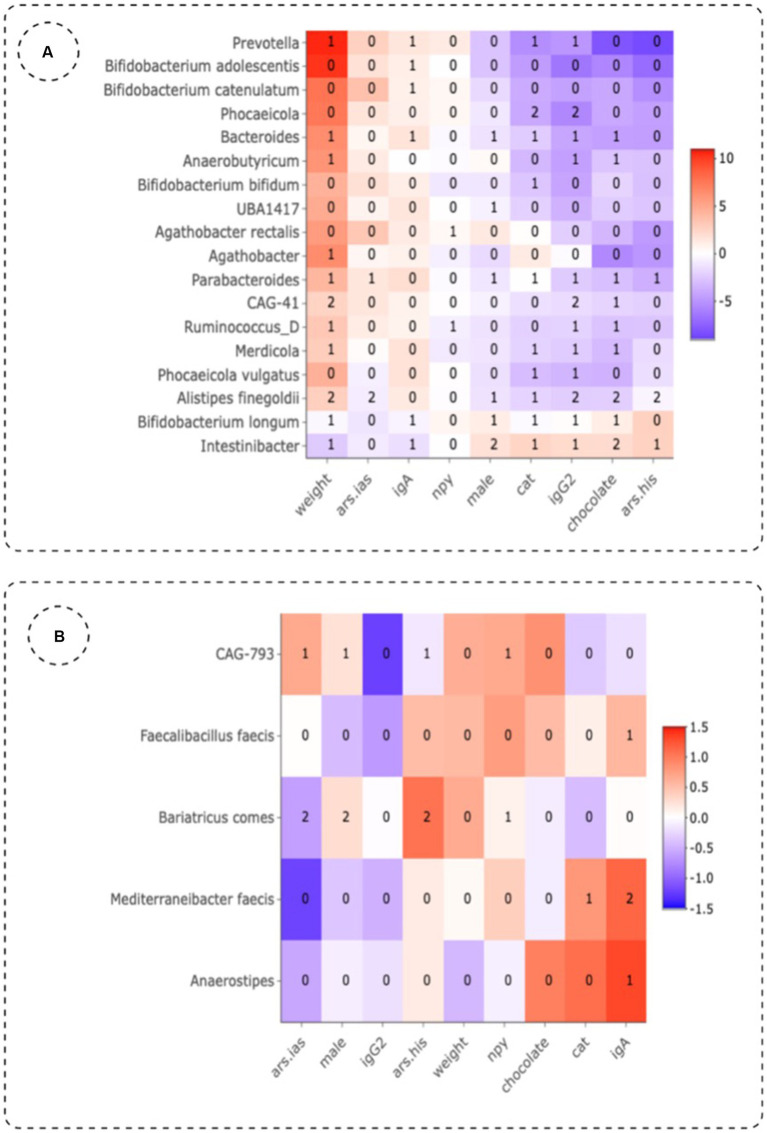
Effect sizes of different variables in the group treated with PBE **(A)** and MPH **(B)** as compared to the placebo group using three differential abundance analysis methods. Numbers in the plot indicate the number of methods that show significant (*p*-value <0.01, adjusted for multiple testing) abundant taxa. Ars.his: hyperactivity score given by teachers on the ADHD-Rating Scale; ars.ias: inattention score given by teachers on the ADHD-Rating Scale; cat: catalase; IgA, immunoglobuline A; IgG_2_, immunoglobuline G_2_; NPY, neuropeptide Y.

Besides the impact of different treatments on the gut microbiome, we also aimed to explore the role of gender, biological markers and ADHD symptoms. First of all, it is important to mention that a steady significant reduction (Wilcoxon test; *p* = 0.0021) of weight (Figure 1B) was observed in the group treated with MPH, which could be linked to a reduced appetite as demonstrated in previous research work (45). PERMANOVA analysis with the adonis2 function of the R package ‘vegan’, using 999 permutations and stratification by subject showed that treatment (placebo, MPH and PBE) of the participants (adjusted-*p*-value = 0.011) explained 3.94% of the variation of the distribution of taxa (data not shown). Sex (adjusted-p-value = 0.016) explained around 1.96% of the variation in microbiome and difference in weight between start and end of the trial (adjusted *p*-value = 0.015) 2% variation. Analyses of the association of different factors with the microbiome and the effect sizes for these covariates on specific taxa (lower/higher abundance) are denoted in Figure 5A for the PBE group and in Figure 5B for the group treated with MPH, both compared to the placebo group. In the PBE group, the following associations between abundance levels of several taxa and the oxidative stress and immune system related biomarkers were observed: high CAT (−4.116) and IgG_2_ levels (−5.692) were negatively associated with abundance levels of *Phocaeicola*, high IgG_2_ levels were negatively associated (−1.654) with abundance levels of *Firmicutes bacterium CAG-41* (Firmicutes) and high IgG_2_ levels (−2.388) were linked to lower abundance of *Alistipes finegoldii* (Bacteroidetes). A high weight (2.474) was linked to higher abundance levels of *Firmicutes bacterium CAG-41* (Firmicutes) as well as on the abundance of *Alistipes finegoldii* (2.737). High behavioral scores ARS-HIS (−0.534) and ARS-IAS (−0.824) were negatively associated with abundance of *Alistipes finegoldii.* Also, more chocolate intake (−2.272) was linked to lower levels of *Alistipes finegoldii,* whereas the abundance of *Intestinibacter* was positively affected by it (2.052). A male gender type (1.702) was also linked to higher levels of *Intestinibacter.* A few significant differentially abundant taxa in the MPH group were observed, but all with a rather low effect size. High levels of the IgA immune biomarker (1.136) were positively associated with the abundance levels of *Mediterraneibacter faecis.* Abundance of *Bariatricus comes* was negatively associated with high ARS-IAS (−0.613) and positively by ARS-HIS scores (1.049). Also, male gender type (0.264) was linked to higher levels of *Bariatricus comes.*

## 4 Discussion

Emerging evidence suggests that the gut microbiota play a pivotal role in the gut–brain axis by influencing metabolism, inflammation, the hypothalamic–pituitary–adrenal axis and neurotransmission (48). Moreover, an association between the gut microbiota and several neuropsychiatric conditions including ADHD has been demonstrated (49, 50). Though ADHD is the most prevalent neurodevelopmental disorder in children and adolescents, current therapy with psychostimulants is far from optimal and there is still insufficient information on the correlation between the mechanisms involved and the microbiome. Nevertheless, emerging evidence suggests that dietary polyphenols could be beneficial in the treatment of oxidative stress related diseases such as ADHD due to their antioxidant and anti-inflammatory properties, while they also could exert a prebiotic effect on gut microbiota (51).

Compositional analysis revealed that *Agathobacter, Alistipes, Bacteroides, Bifidobacterium, Cryptobacteroides, Faecalibacterium, Gemmiger, Phocaeicola, Prevotella, Romboutisa* and *Rumminococcus_E* were the most abundant genera at baseline. Interestingly, *Bifidobacteria* make up most part of the overall relative abundance in our study population at baseline. Generally, at birth and during early development, bifidobacterial populations are found to be the most abundant genus and abundance levels slowly decrease considerably but remain relatively stable during adulthood, futher decreasing at old age (52, 53). Moreover, various studies have identified the genus *Bifidobacterium* as having a potential relevance to ADHD albeit with contradictory outcomes. According to a systematic review, some studies found a nominal increase in *Bifidobacterium* in ADHD compared to control subjects while others stated that the abundance of *Bifidobacterium* is reduced in ADHD (15, 54). This increase in *Bifidobacterium* in the ADHD cohort can occur at the expense of more developmentally appropriate bacteria (i.e., Bacteroidetes) with its dominance during childhood and possible deficiency in early life (54). Though in our study set-up no healthy controls were included, our results show high relative abundance of *Bifidobacterium* in pediatric ADHD patients which can be linked to an enhanced synthesis of the dopamine precursor phenylalanine of which high levels have been linked to ADHD symptoms (55). Also, at genus level, a lower abundance of *Faecalibacterium* could be observed in our baseline study population, which is in line with earlier research reporting lower levels in ADHD patients as compared to healthy controls (56). *Faecalibacterium* are found to exhibit anti-inflammatory properties and can thus be beneficial for ADHD patients since increased levels of pro-inflammatory markers have been associated with ADHD. Alterations in abundance levels of *Faecalibacterium* may thus play a role in the etiology and/or symptomatology of ADHD (57).

Looking at phylum level, no clear trend toward altered F/B-ratio could be observed for the three study cohorts at 10-weeks compared to baseline. Since the F/B ratio at baseline was already different between the treatment groups and individual patients appeared to fluctuate both up and down in F/B-ratio, no statistical significance was obtained. Moreover, since the F/B ratio might already be elevated at baseline in our study cohort, a significant increase in ratio as a result of a 10-week treatment might therefore remain undetected as well as a possible modulation of the F/B ratio by PBE.

Bacterial diversity analyses at family level did not reveal significant differences in any of the treatment groups. Though no statistical significance could be obtained, results of the PCoA plot suggest that there are some samples with unique factors in the populations not treated with PBE (placebo and MPH) which are absent in the samples treated with PBE. Based on our findings, we cannot conclude that PBE exerts a prebiotic effect on the gut microbiome. Nevertheless, we did notice a difference in abundance level of taxa (*UBA-932* and *CAG-272*) in some patients treated with PBE. In fact, these individuals could thus be responders to PBE therapy. This would be in line with our other research findings demonstrating a significant decrease in total ADHD-RS as rated by teachers and thus an improvement of ADHD behavior (22). Yet, qPCR should confirm these results and can maybe even highlight prebiotic effects in the other patients treated with PBE. In addition, according to Stiernborg et al. (58), psychostimulant use in ADHD children is associated with significant microbial alterations between children on ADHD medication as compared to non-medicated ADHD patients. This study found that ADHD children on psychostimulant drugs including MPH had a significantly different taxonomic β-diversity. Moreover, they found a lower abundance of *Bacteroides stercoris CL09T03C01* and bacterial genes encoding an enzyme in vitamin B_12_ synthesis. Despite the fact that these novel findings still need to be replicated in other studies to reveal the causal relationships between gut microbiota and ADHD, our study has been unable to demonstrate the impact of MPH on gut microbial composition. This discrepancy could be attributed to our limited number of patients as opposed to the sample size used in the research by Stiernborg et al.

Our results thus suggest that none of the treatments corresponds with large scale changes in community composition of the human fecal microbiome content during this 10-week randomized trial. This in line with previous findings of Stevens et al. (59) who found that micronutrient treatment consisting of a blend of vitamins, minerals, amino acids and antioxidants did not drive large-scale changes in microbial composition. Possibly, supplementation of 10 weeks is not long enough to observe these substantial changes in gut microbiome. In addition, apart from medication use such as antibiotic therapy, no specific inclusion criteria regarding their effects on gut microbioma were implemented in our study. For instance, the study population was not screened for dietary habits (e.g., vegetarian or vegan) neither for defecation frequency nor lifestyle habits. Taken together the possible large differences between the individuals at the start regarding microbial composition due to differences in diet and lifestyle as well as the short supplementation period hampers to find significant results. Nevertheless, collecting personal data and information and analysis of biomarkers in blood samples, allowed us to perform an in-depth analysis of covariates. Several co-variates were associated with the microbial constellation.

The impact of several environmental variables on microbial communities was investigated. Treatment showed the largest explanatory value for microbiome variation in our study. Though our analyses revealed that this effect size was rather small (covariate accounted for 3.94%) it was highly significant. Nevertheless, it only explained a small part of genus abundance variation, suggesting additional contribution from other factors such as environment or genetic background. Our findings are in line with previous research work which also showed that additional factors (e.g., use of medication and/or antibiotics and consumption of pre-or probiotics) have a profound impact on microbiome composition (60, 61). Moreover, it should be taken into account that there is also therapeutic evidence regarding the impact of eating patterns and dietary interventions on gut microbial composition (62). Though multiple factors are known to affect gut microbial composition, collection of all this valuable information is considered a strength of this study.

Another strength of our study is the supplementation with a standardized extract, complying with United States Pharmacopeia (USP) requirements regarding polyphenolic constituents and procyanidin content, to make sure the bioavailability of PBE is sufficient enough to notice differences (63). Moreover, it has been demonstrated that microcrystalline cellulose does not induce microbial shifts and placebo supplementation therefore does not alter the gut microbiome itself (64).

In general, the interactions between polyphenols and gut microbiota are reciprocal (65). Research not only demonstrated that polyphenols lead to modulation of the gut microbial composition, but also that the gut microbiota plays an important role in the biotransformation of polyphenolic compounds (24, 65). The gut microbiota thus contributes to the metabolism of dietary polyphenols, thereby impacting the bioavailability of both parent polyphenols and their (potentially bioactive) metabolites (66, 67). Further research within our research group is therefore imperative to improve our understanding of this two-way interaction by investigating the biotransformation processes of ingested PBE in the presence of intestinal microbiota (68). This longitudinal intervention study already offers some important new insights in how PBE influences microbial composition and may thus offer valuable information on which microbial strains may affect biotransformation processes of PBE.

In conclusion, only small changes in the gut microbiome of the participants of either treatment group were noticeable. This could be due to the small sample size per group and therefore a rather low power, and a high background noise since the gut microbiome of children at this age is very flexible. Moreover, weight loss was observed in the group treated with MPH (45), thereby increasing the complexity of interpretation of the analysis. Further research involving more participants, a longer supplementation period and possibly more sampling points is required to establish potential therapeutic efficiency of PBE in gut microbial modulation.

## Data availability statement

The datasets presented in this study can be found in online repositories. The names of the repository/repositories and accession number(s) can be found at: https://www.ebi.ac.uk/ena, PRJEB65903.

## Ethics statement

The studies involving humans were approved by EC 15/35/365 (University Hospital Antwerp) 2016/0969 (University Hospital Ghent) EC approval 4,656 (Hospital Network Antwerp). The studies were conducted in accordance with the local legislation and institutional requirements. Written informed consent for participation in this study was provided by the participants’ legal guardians. The studies were conducted in accordance with the local legislation and institutional requirements. Written informed consent for participation in this study was provided by the participants’ legal guardians/next of kin. Written informed consent was obtained from the minor(s)’ legal guardian/next of kin for the publication of any potentially identifiable images or data included in this article.

## Author contributions

A-SW: Investigation, Writing – original draft, Writing – review & editing. SA: Writing – review & editing. TR: Formal analysis, Writing – review & editing. TB: Writing – review & editing. SL: Resources, Writing – review & editing. NH: Resources, Supervision, Writing – review & editing.
