# Supplementary material for: Enhancing pediatric attention-deficit hyperactivity disorder treatment: exploring the gut microbiota effects of French maritime pine bark extract and methylphenidate intervention
Source: Front Nutr. 2024 Aug 27;11:1422253. doi: 10.3389/fnut.2024.1422253 (PMC11385872; doi:10.3389/fnut.2024.1422253)
Supplement: Supplementary file 1 [file Table_1.DOCX]

Supplementary Material


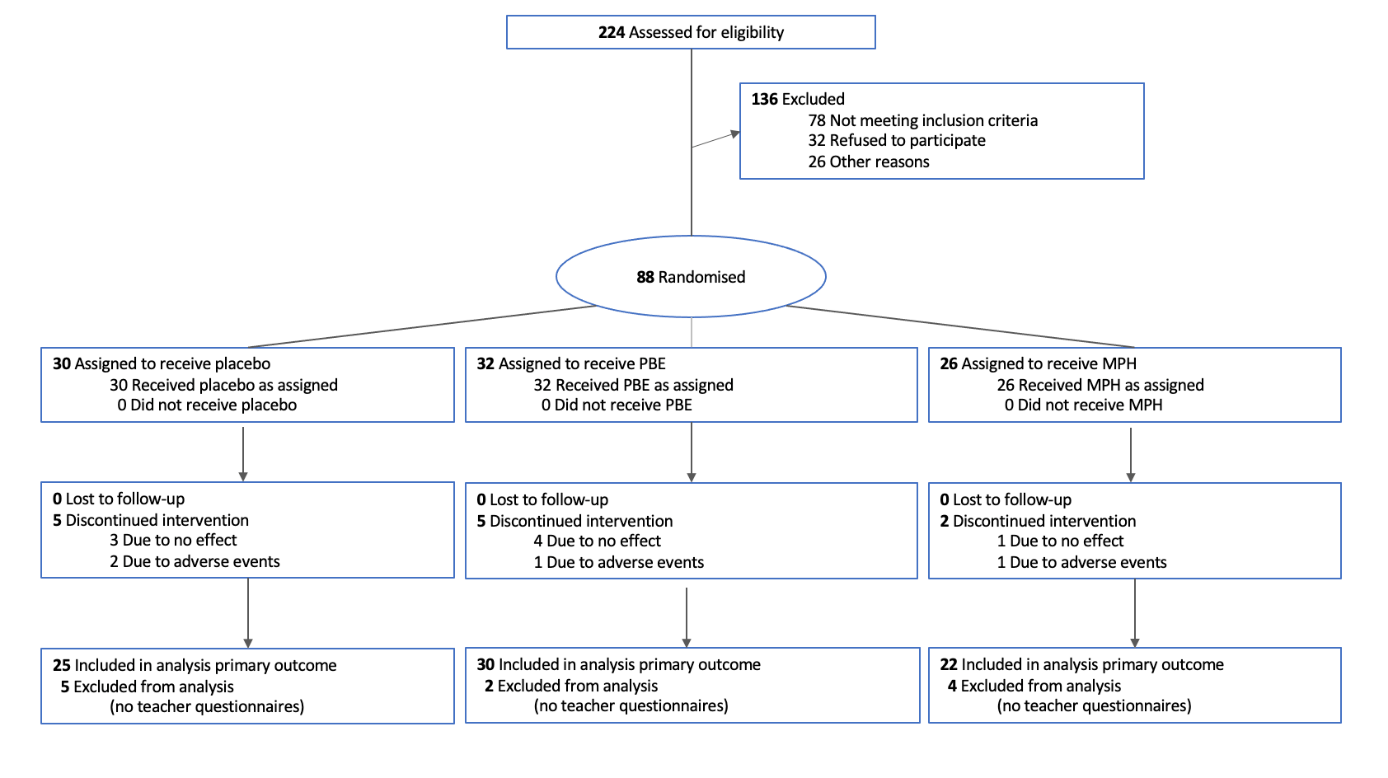


Figure S1. CONSORT flow chart of the clinical trial. MPH: Methylphenidate; PBE: French Maritime Pine Bark Extract.


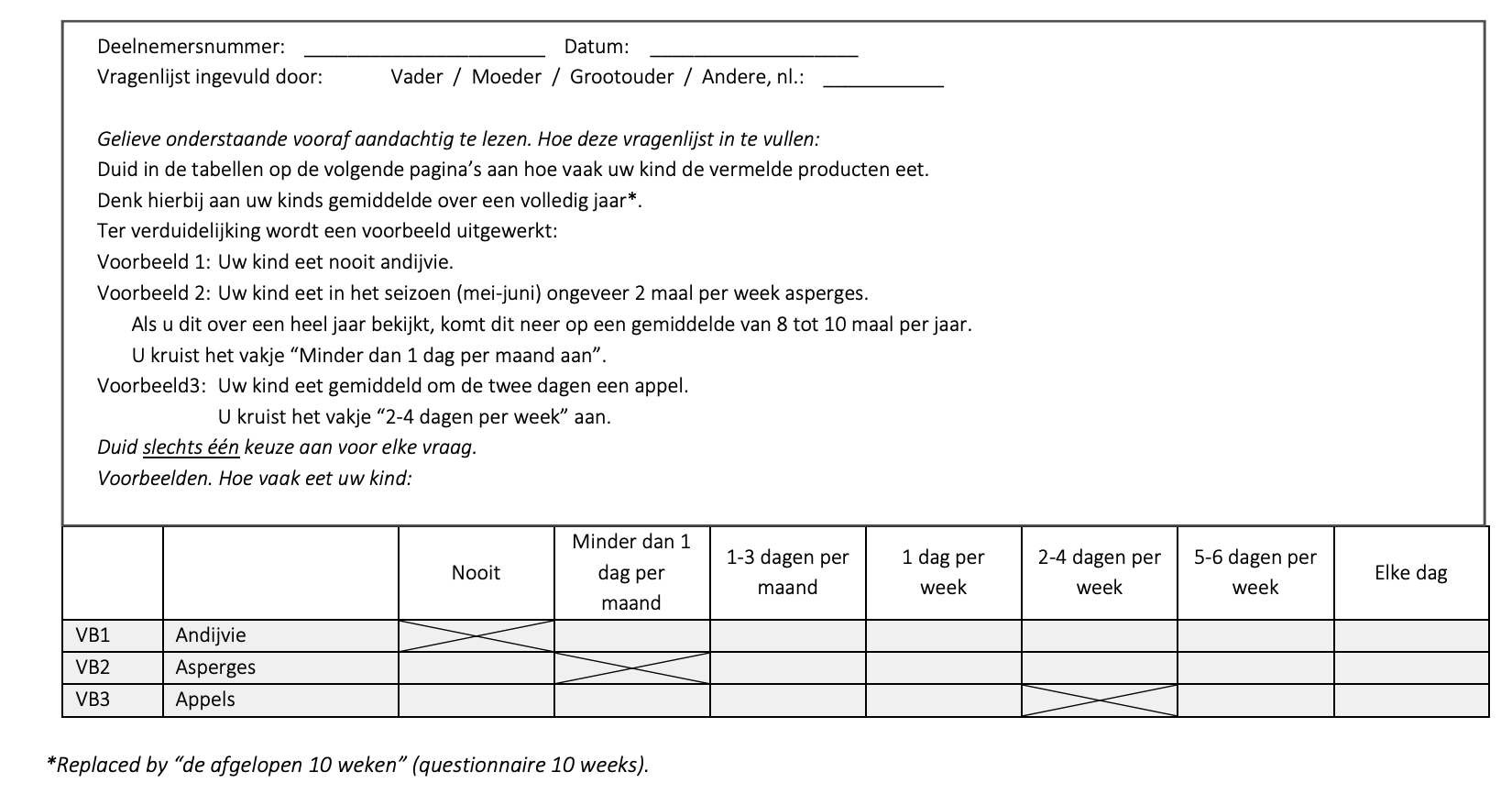


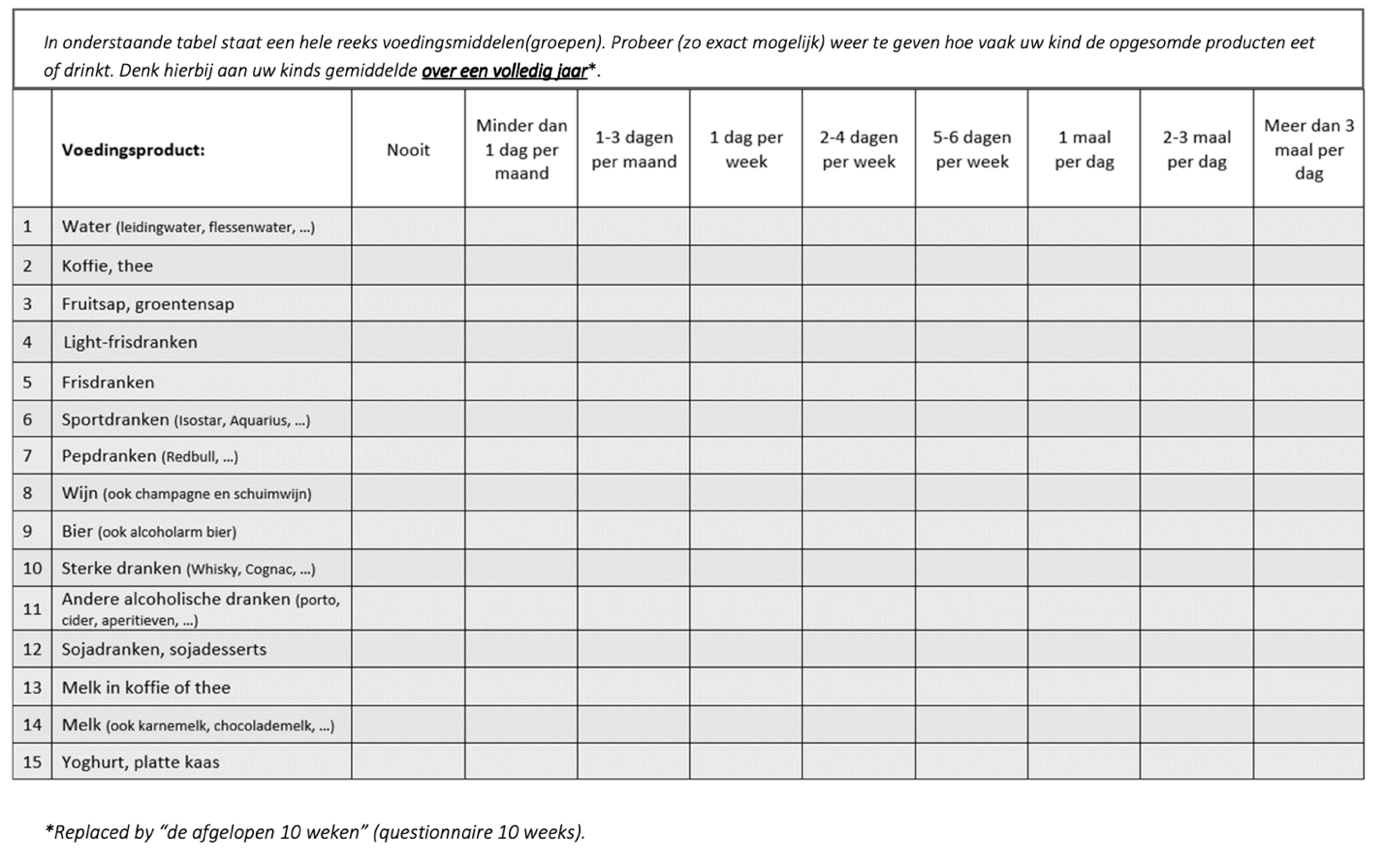


**
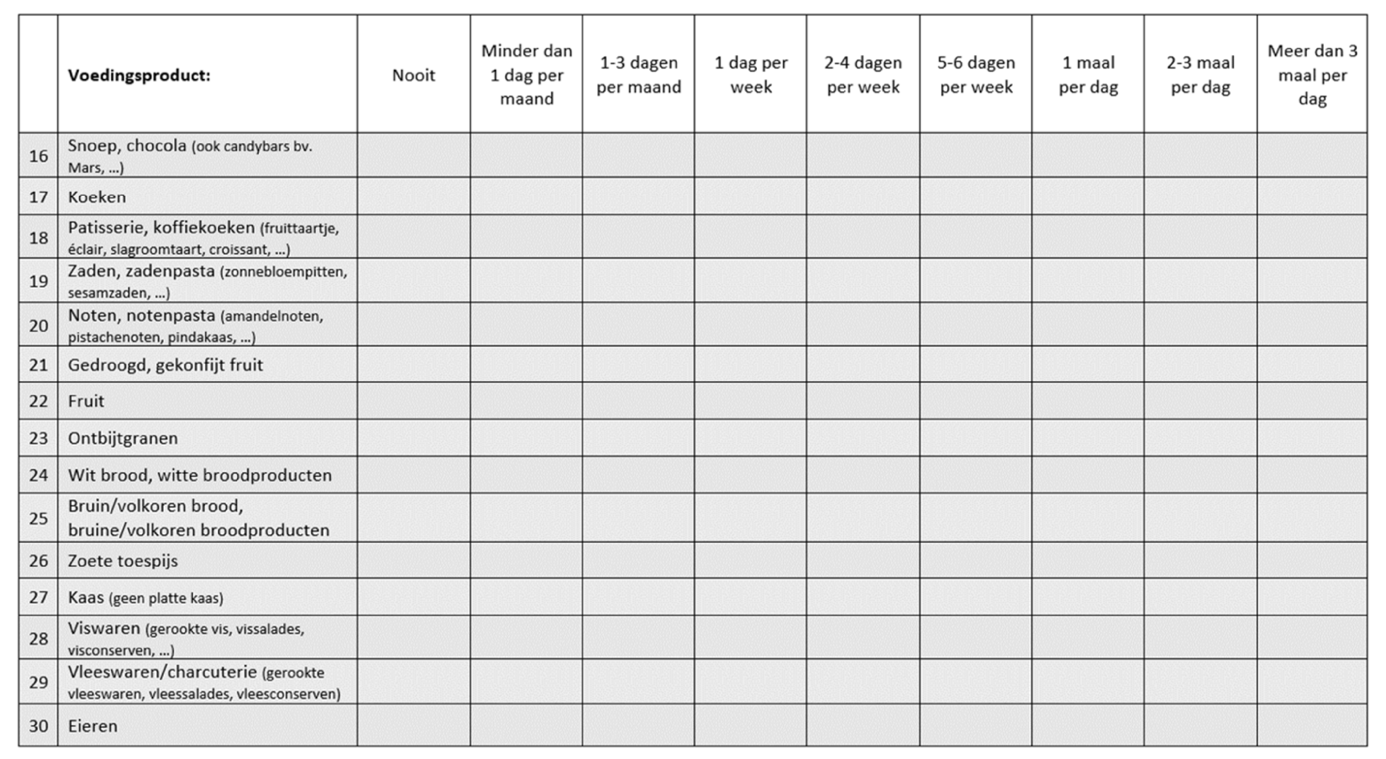
**


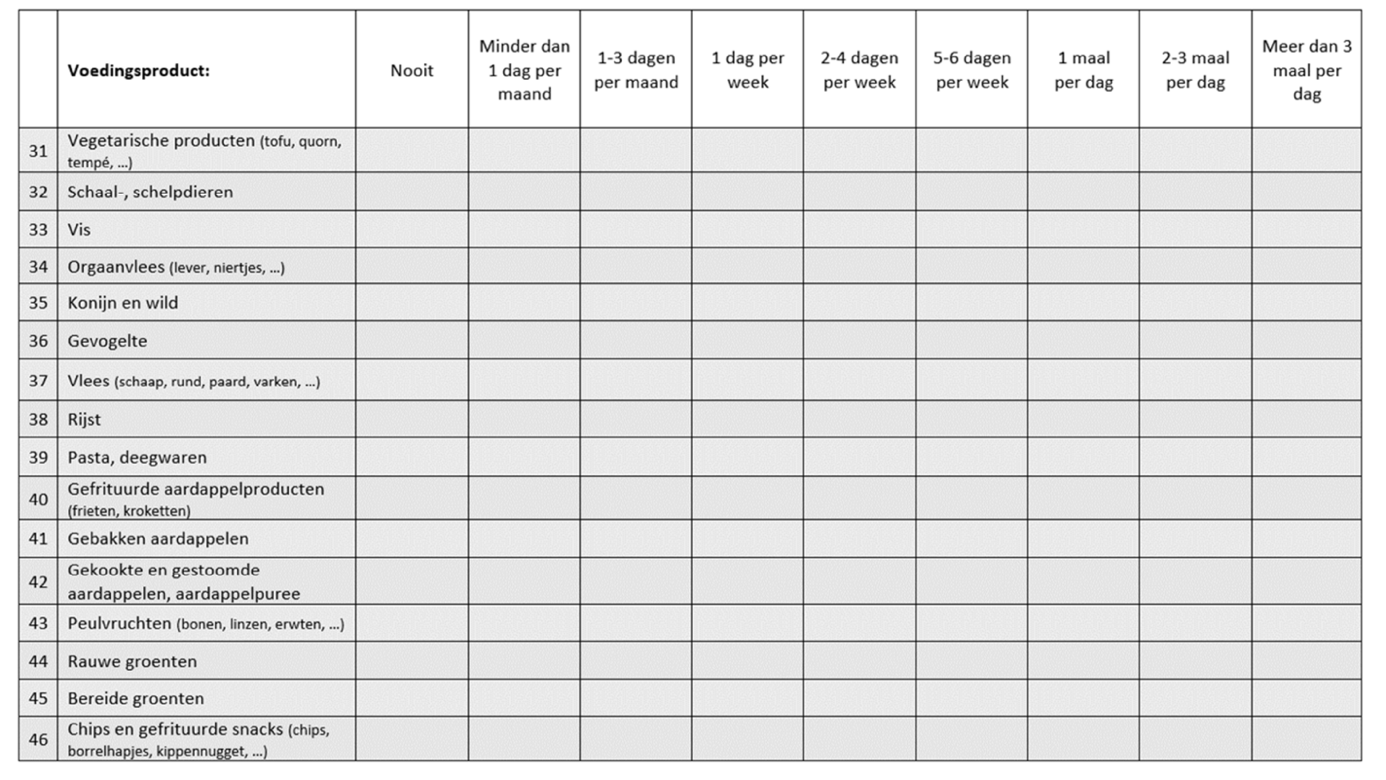


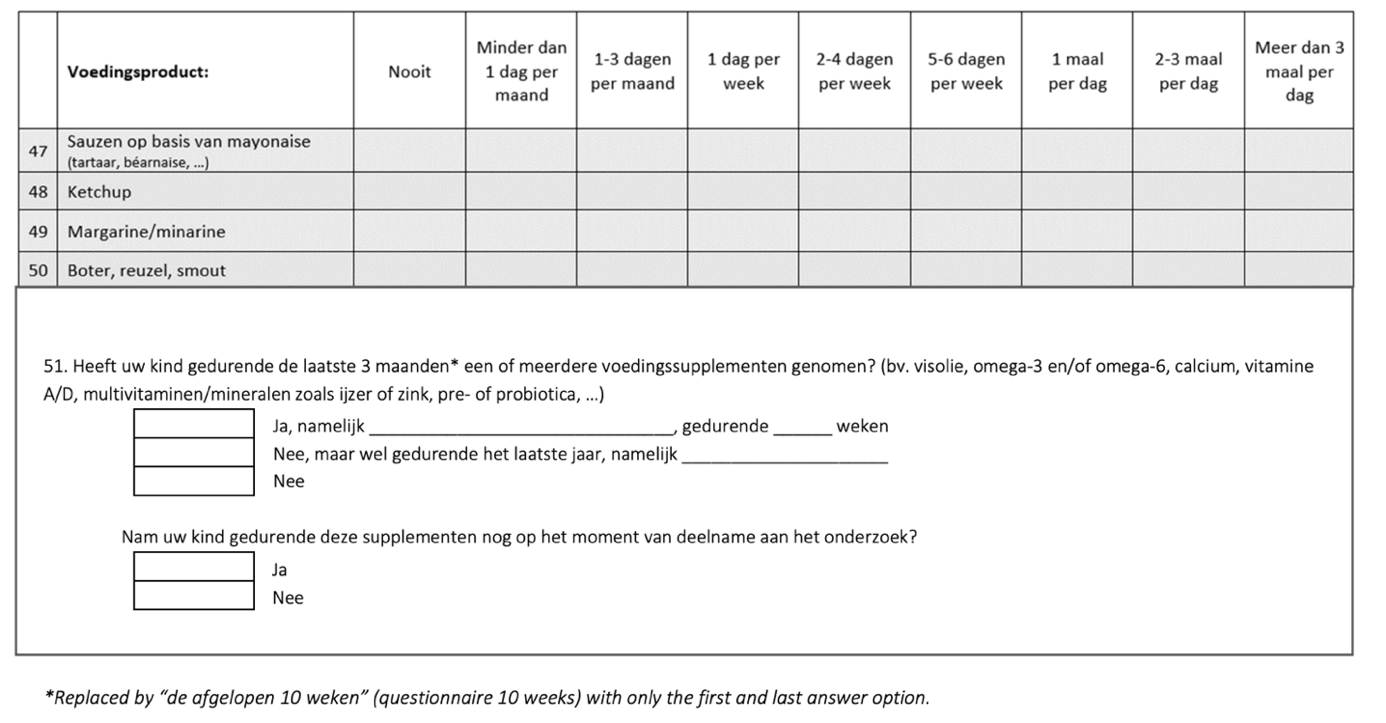


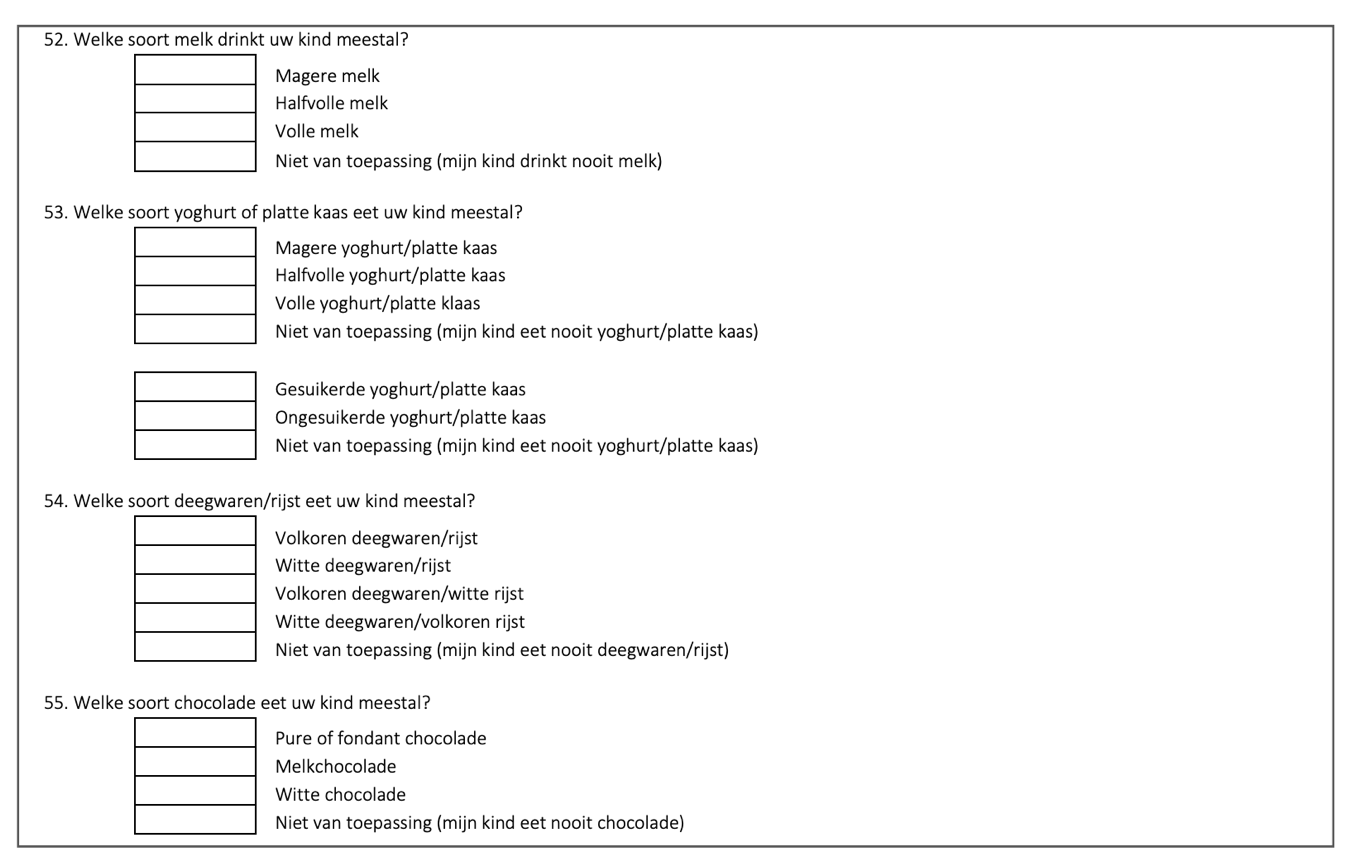


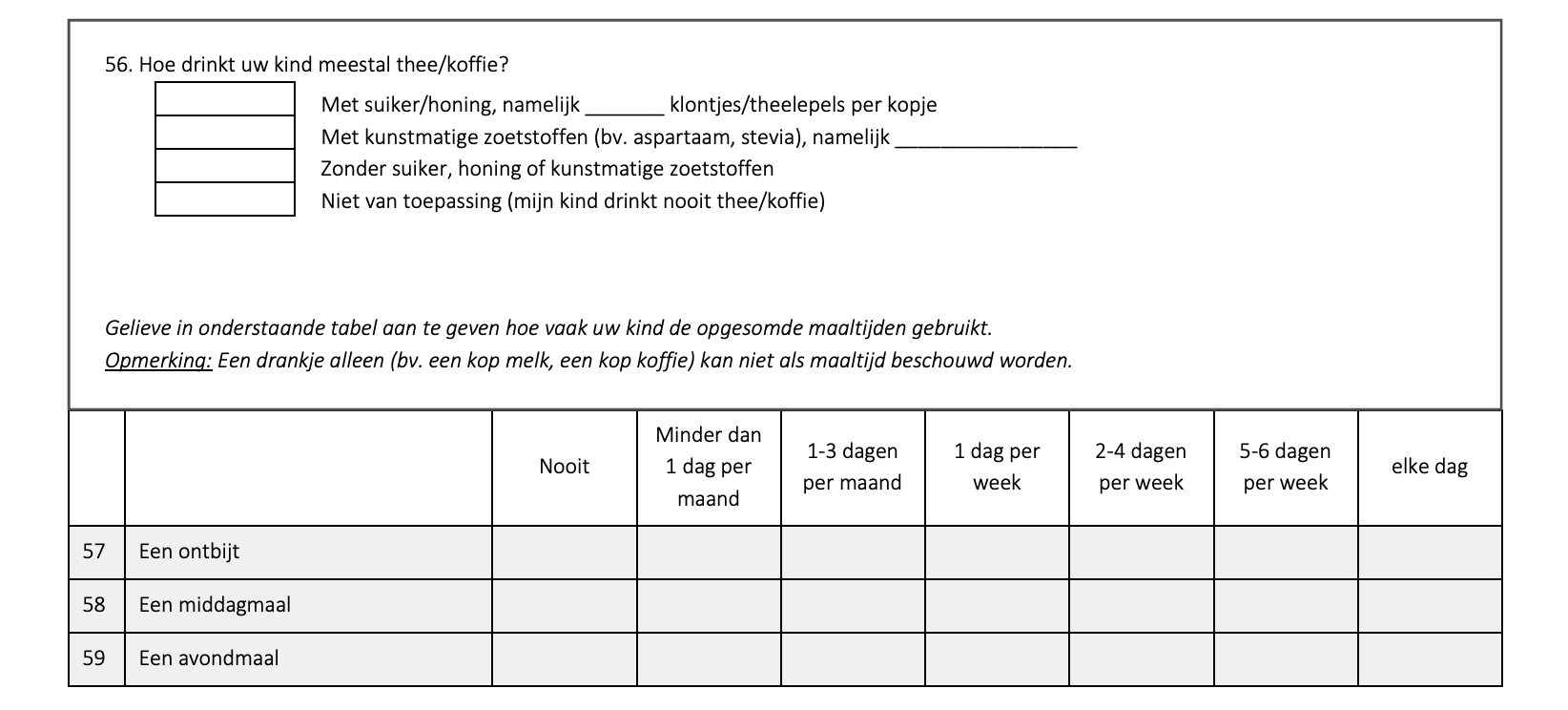


**Figure S2**. Food Frequency Questionnaire (FFQ) filled out by participants.


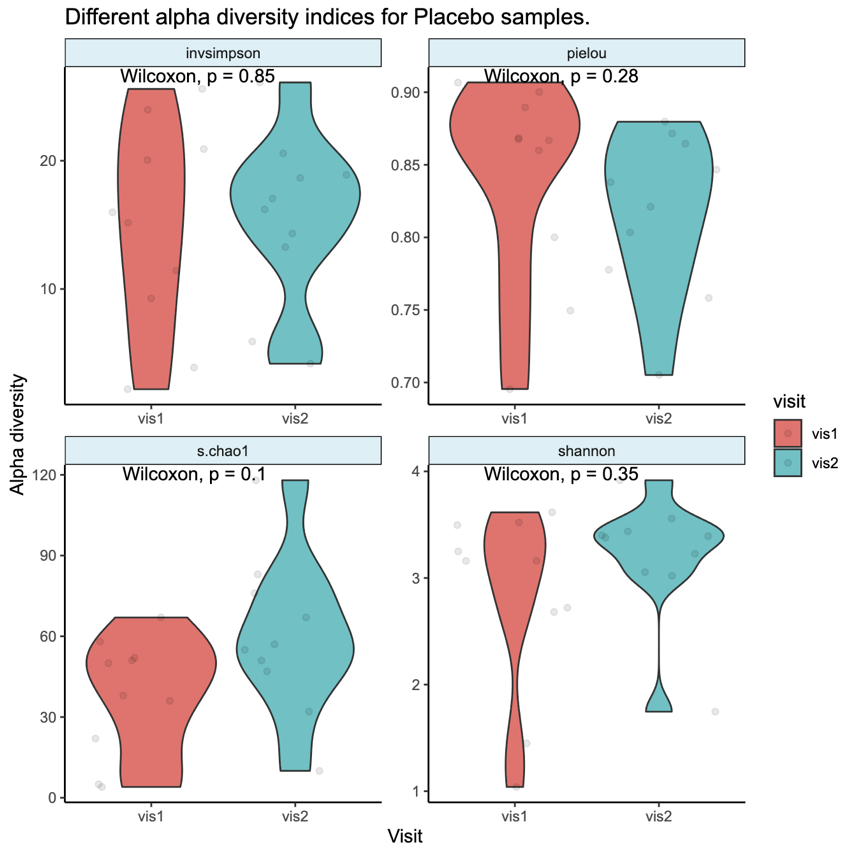


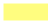


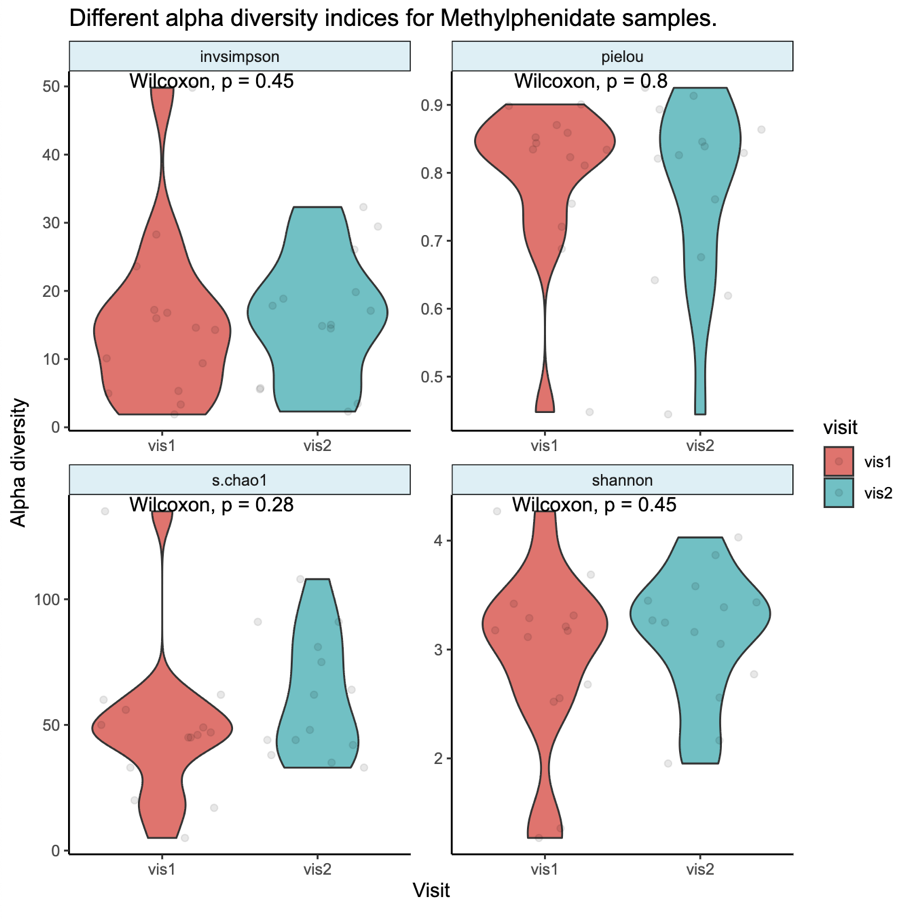

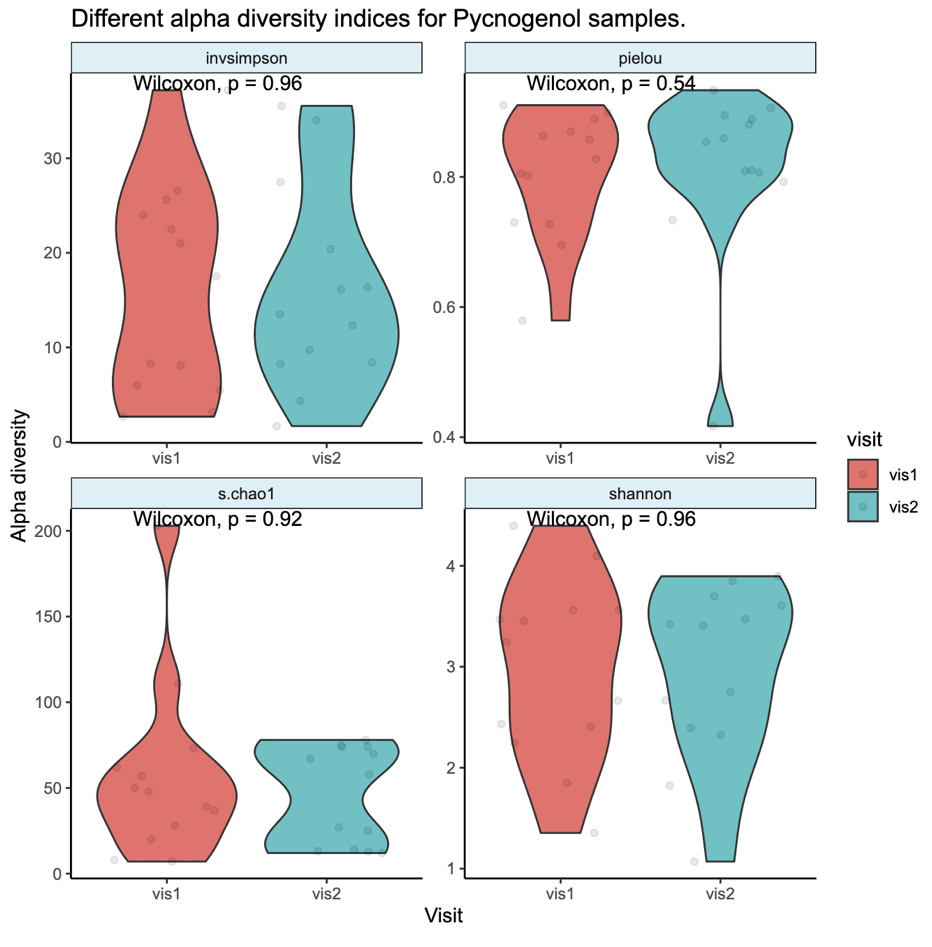


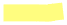

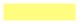


Figure S3: Overview of alpha diversity indices for the three different treatment groups (placebo, methylphenidate and Pycnogenol (PBE)).


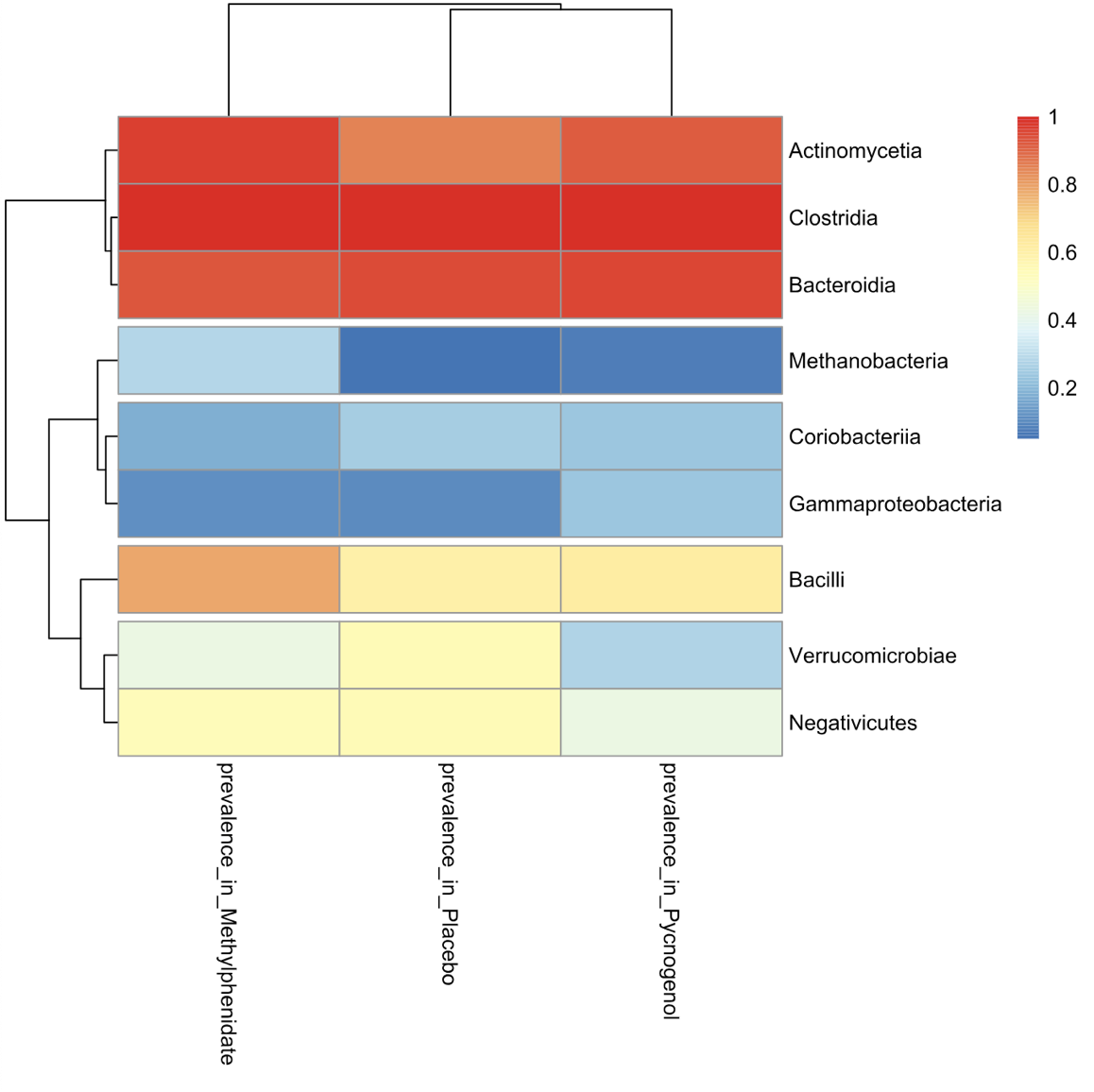


Figure S4: Heatmap representing the core microbiome of the included patients per treatment group at class taxonomical level. The prevalence (indicated by the color code; expressed in %) shows in how many samples a certain taxon is present. The taxa are shown if they are present at least in 10% in one of the treatment groups.


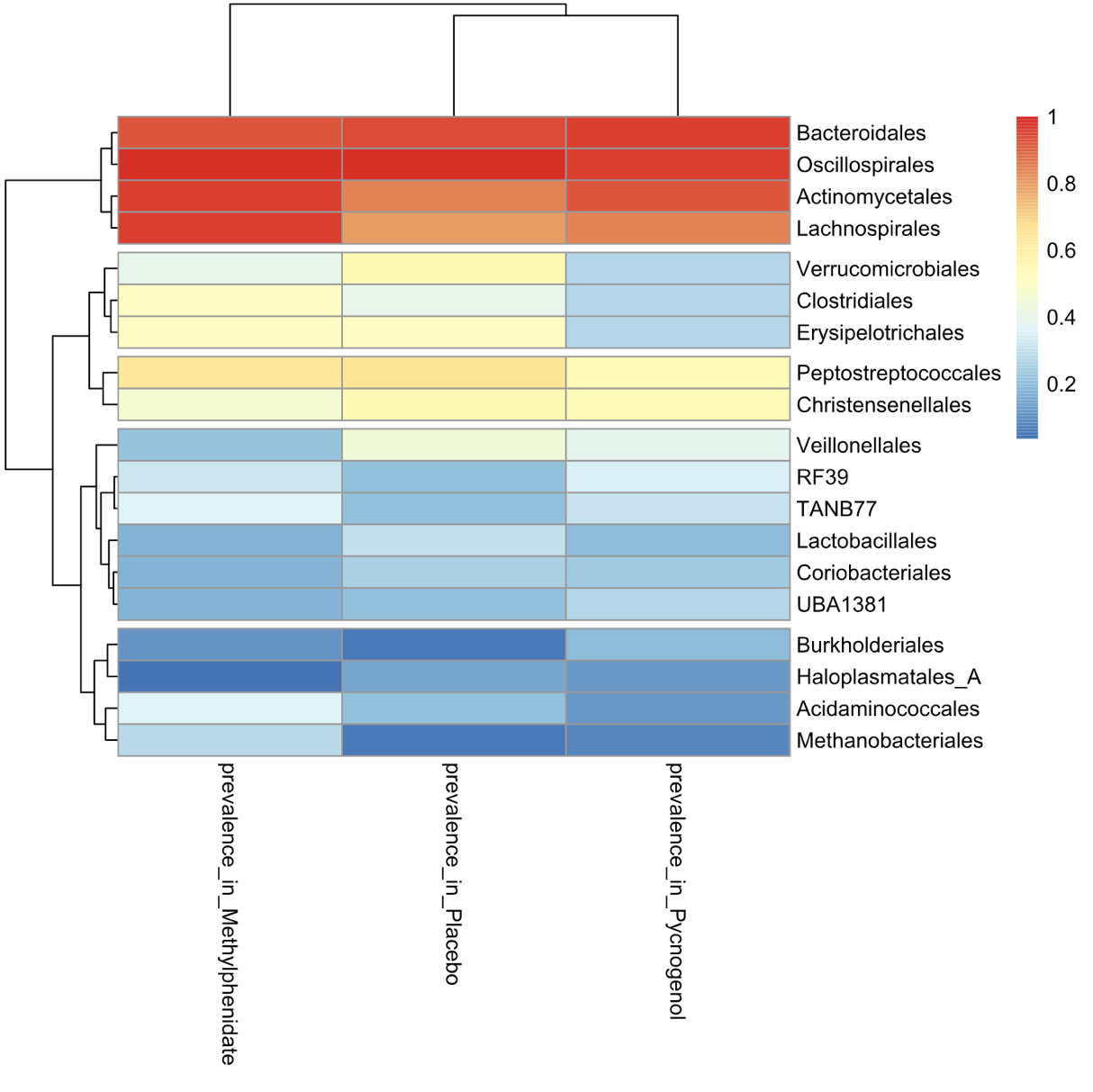


Figure S5: Heatmap representing the core microbiome of the included patients per treatment group at order taxonomical level. The prevalence (indicated by the color code; expressed in %) shows in how many samples a certain taxon is present. The taxa are shown if they are present at least in 10% in one of the treatment groups.


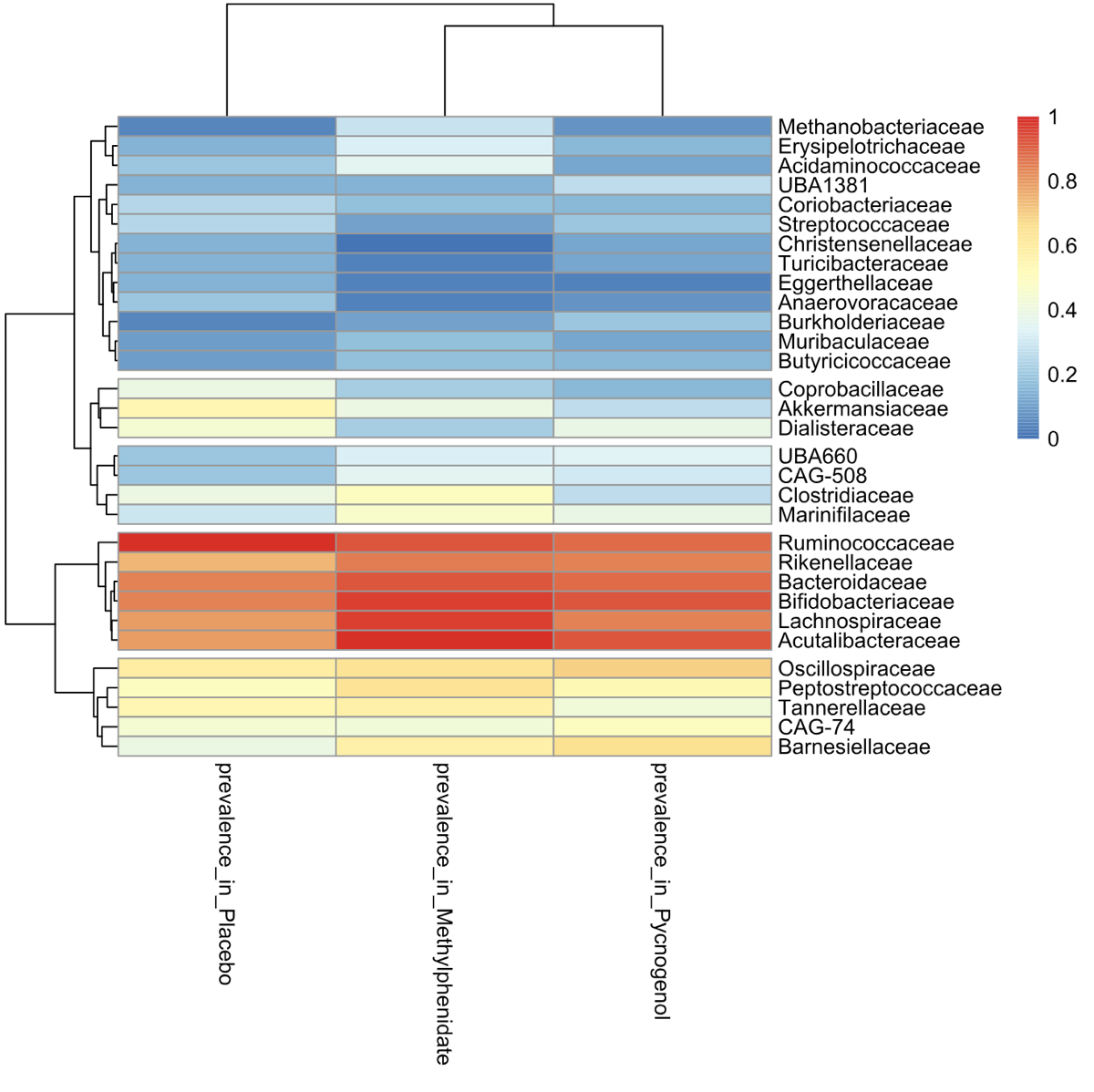


Figure S6: Heatmap representing the core microbiome of the included patients per treatment group at family taxonomical level. The prevalence (indicated by the color code; expressed in %) shows in how many samples a certain taxon is present. The taxa are shown if they are present at least in 10% in one of the treatment groups.


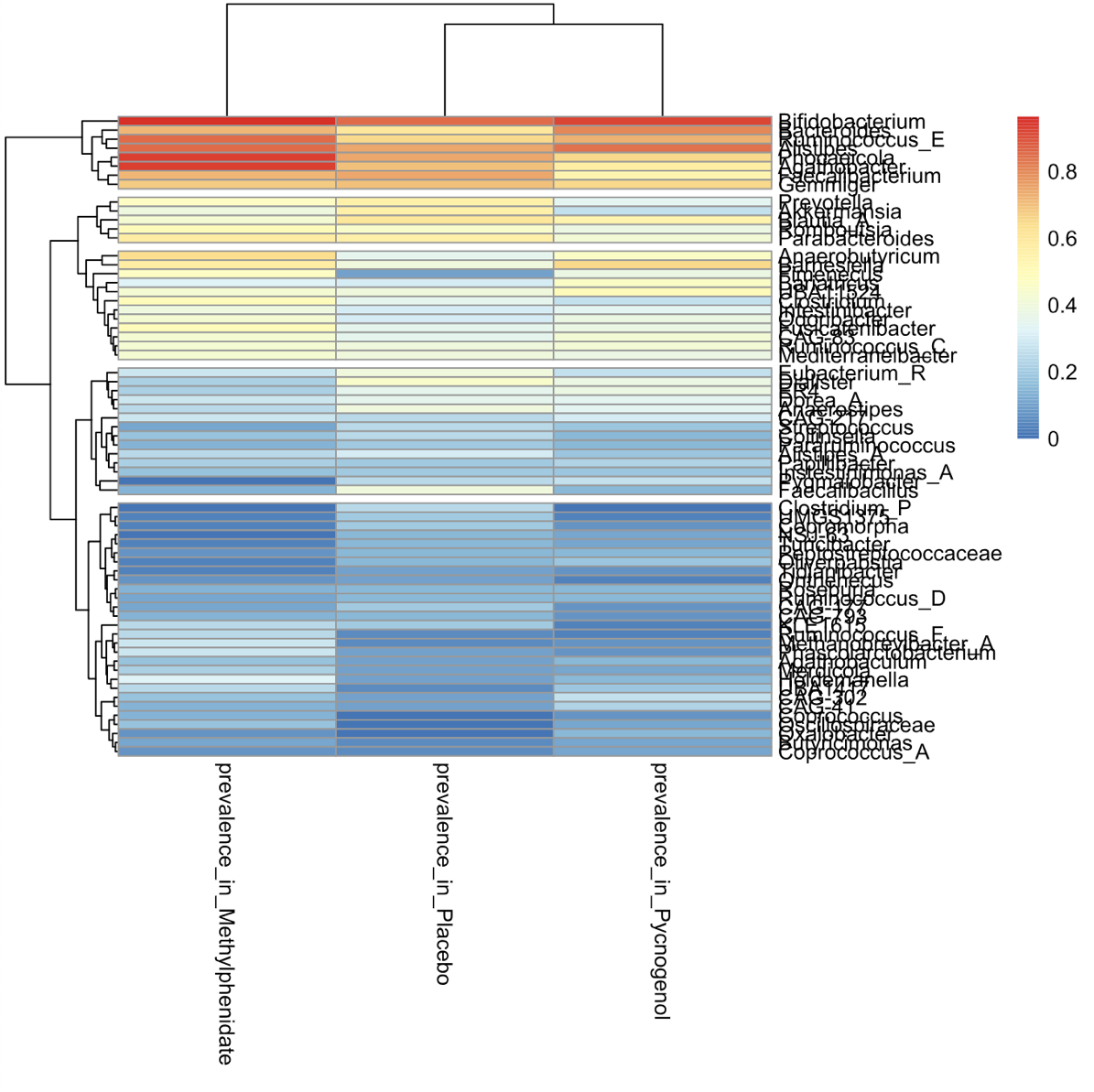


Figure S7: Heatmap representing the core microbiome of the included patients per treatment group at genus taxonomical level. The prevalence (indicated by the color code; expressed in %) shows in how many samples a certain taxon is present. The taxa are shown if they are present at least in 10% in one of the treatment groups.
